# SNP and Mutation Data on the Web – Hidden Treasures for Uncovering

**DOI:** 10.1002/cfg.131

**Published:** 2002-02

**Authors:** Michael R. Barnes

**Affiliations:** Genetic Bioinformatics GlaxoSmithKline Pharmaceuticals New Frontiers Science Park (North) Third Avenue Harlow Essex CM19 5AW UK

## Abstract

SNP data has grown exponentially over the last two years, SNP database evolution has matched this growth, as initial development of several independent SNP databases has given way to one central SNP database, dbSNP. Other SNP databases have instead evolved to complement this central database by providing gene specific focus and an increased level of curation and analysis on subsets of data, derived from the central data set. By contrast, human mutation data, which has been collected over many years, is still stored in disparate sources, although moves are afoot to move to a similar central database. These developments are timely, human mutation and polymorphism data both hold complementary keys to a better understanding of how genes function and malfunction in disease. The impending availability of a complete human genome presents us with an ideal framework to integrate both these forms of data, as our understanding of the mechanisms of disease increase, the full genomic context of variation may become increasingly significant.


**Comparative and Functional Genomics**
*Comp Funct Genom* 2002; **3**: 67–74.
**DOI:** 10.1002/cfg.131

**Review**

### SNP and mutation data on the Web – hidden treasures for uncovering.

Michael R Barnes*
*Genetic Bioinformatics, GlaxoSmithKline Pharmaceuticals, New Frontiers Science Park (North), Third Avenue, Harlow, Essex CM19 5AW, UK*

*Correspondence to:
Genetic Bioinformatics, GlaxoSmithKline Pharmaceuticals, New Frontiers Science Park (North), Third Avenue, Harlow, Essex CM19 5AW, UK.
E-mail:
Michael_R_Barnes@gsk.com

Received: 1 November 2001
Accepted: 21 November 2001
Published online:
12 December 2001

### Abstract

**SNP data has grown exponentially over the last two years, SNP database evolution has matched this growth, as initial development of several independent SNP databases has given way to one central SNP database, dbSNP. Other SNP databases have instead evolved to complement this central database by providing gene specific focus and an increased level of curation and analysis on subsets of data, derived from the central data set. By contrast, human mutation data, which has been collected over many years, is still stored in disparate sources, although moves are afoot to move to a similar central database. These developments are timely, human mutation and polymorphism data both hold complementary keys to a better understanding of how genes function and malfunction in disease. The impending availability of a complete human genome presents us with an ideal framework to integrate both these forms of data, as our understanding of the mechanisms of disease increase, the full genomic context of variation may become increasingly significant. Copyright © 2001 John Wiley & Sons, Ltd.**

**Keywords:** **bioinformatics; genetics; human genome; SNP; Mutation; databases**

### Introduction

As the sequencing of the human genome has drawn into its final stages, focus on human genetic variation has come to the fore – mainly in the form of single nucleotide polymorphisms (SNPs) – which offer to revolutionise the way we study human genetics and disease. For the geneticist, SNP markers are a key research material for genetic association with heritable traits, but for those with a wider interest in genomics and biology, the new SNP data is also a valuable resource. The variation we see so far can tell us many things about the functional parameters and critical regions of a gene, protein, regulatory element or genomic region. More specifically knowledge of other forms of human variation such as human mutation can tell us a great deal about the function of genes and biological pathways by studying their dysfunction in genetic disease.

SNPs are the commonest form of variation in the genome, comparison of any two chromosomes will generally reveal SNPs at 1.2 kb average intervals across the genome [1]. SNPs as disease markers are now the great hope of genetics, but this focus has not always been so, despite their abundance in the genome, without knowledge of genome sequence, SNP identification is a laborious process which has made SNP availability limited. Instead geneticists used more easily identified, but less plentiful, tandem repeat sequences (microsatellites) as markers. These have been widely used for linkage analysis of family based disease inheritance patterns which because of family relatedness can extend over many megabases. Such family based linkage scans have been very successful in mapping mutations causing single gene disorders or Mendelian traits, but have been largely unsuccessful in detecting the multiple genes responsible for common complex diseases [12]. An alternative approach for mapping complex disease genes is to use markers to detect population based allelic association or linkage disequilibrium between markers and disease alleles. These associations can be very strong even where the corresponding family linkage signal is weak or absent. The drawback to this approach is that population based association usually extends over much shorter genomic distances anywhere between 5–100 kilobases [10]. Detection of this association demands a massive increase in marker density with more than 500 000 markers estimated to be needed

Copyright © 2001 John Wiley & Sons, Ltd.

to cover the genome for an association scan compared to the 200–500 markers needed for a family based linkage scan.

SNP markers are probably the only viable option for these population based studies, but until very recently demand has completely outstripped SNP availability. Ambitious whole genome SNP association studies simply could not be attempted with available markers. This situation has now changed – the completion of the first draft of the human genome has spawned several large-scale SNP discovery projects – we now have a wealth of SNP data to facilitate these studies. Genetics is now entering an exciting new era, where marker resources and locus information are no longer the main factors limiting the success of complex disease gene hunting, the emphasis now lies on good study design and the best possible study populations. Likewise biologists will benefit from this wealth of data, with a more complete view of variation in genes and regulatory regions.

A strong informatics infrastructure is critical to effectively exploit this data. Databases need to effectively integrate the array of newly generated SNP data with pre-existing human mutation data onto the framework of the human genome. Only then will it be possible to construct sophisticated SNP maps and take into account the full complexity of human genetic variation that causes disease.

### When is a SNP not a SNP?

Some definition of the term SNP is important for this review. In the strictest sense a SNP is a single base change, occurring at a frequency of $>1\%$ – termed a polymorphism. When a single base change occurs at $<1\%$ it is strictly considered to be a mutation. This terminology is often disregarded, many have suggested that 'mutations' occurring at $<1\%$ in general populations should be termed low frequency variants, whereas the term 'Mutation' implicitly suggests a variant with a defined phenotype often inherited in a Mendelian manner. Mutation databases and polymorphism databases have generally been divided by this definition of polymorphisms which are widespread in populations and mutations which are usually rare and are not generally thought to occur widely in populations, but instead occur sporadically or are inherited in a Mendelian manner. A grey area exists, which argues against the rigidity of this division of data (Figure 1). In a heterozygote form some Mendelian mutations have been linked to complex disease susceptibility and indeed are relatively

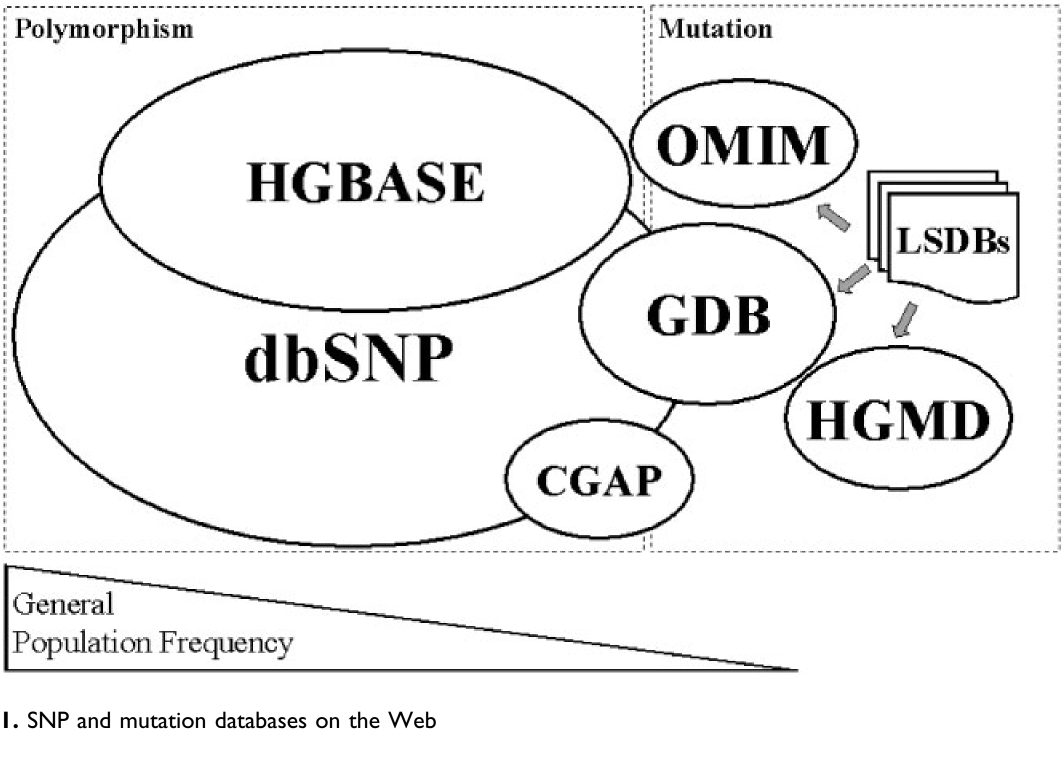

**Figure 1.** SNP and mutation databases on the Web

Copyright © 2001 John Wiley & Sons, Ltd.

widely spread in populations. For example, homozygote mutations in the cystathione beta synthase gene cause homocystinuria, a rare disorder inducing multiple strokes at an early age, the heterozygotes do not share this severe disorder, but do have an increased lifetime risk of stroke [7]. In Caucasians the population frequency of homozygote homocystinuria mutations, is only one per 126 000, but in the same population, heterozygote frequency is relatively high at one per 177. This illustrates the point that it may not always be helpful to separate polymorphism and mutation data, although clearly both forms of data need to be well defined.

SNP databases have progressed greatly in the last year and we are now very close to the ultimate goal of a comprehensive central SNP database, presented to the user in an integrated form across the human genome. Mutation databases are lagging considerably behind in terms of data integration and visualisation and consequently most of this potentially valuable mutation data is not readily accessible to the biologist. The availability of a complete draft of the human genome, finally presents an opportunity to bring these two sources of data together in a complete genomic context, without compromising the integrity of either data collection.

### Single nucleotide polymorphism (SNP) databases

The deluge of SNP data generated over the past two years can primarily be traced to two major sources: The SNP consortium (TSC) [1] and members of the human genome sequencing consortium, particularly the Sanger Institute and Washington University. The predominance of SNP data from this small number of closely related sources has facilitated the development of something very close to a central SNP database – dbSNP at the NCBI [13]. Other valuable databases have developed using this central resource as a reference, these tools and databases bring focus to specific subsets of SNP data, eg., Gene orientated SNPs, while enabling further data integration around dbSNP. A selection of these tools and databases is summarised in Table 1.

### dbSNP – a universal SNP database?

Established in September 1998, dbSNP currently contains 3.9 million SNPs (Build 100 – Nov 2001). These SNPs can be grouped into a non-redundant set of 2.4 million SNPs, known as Reference SNPs (RefSNPs). Approximately 10% of these RefSNPs do not currently map to the draft human genome, which leaves 2.16 million SNPs with immediate utility for genetics. In the wake of the TSC and other SNP discovery projects, further SNP submissions will continue from the genome centres in the final stages of genome finishing, but dbSNP growth is not likely to continue at the rates it has seen in the past two years. Most journals now require SNP submission to dbSNP before publication (a practice which needs to be encouraged), these are estimated to add to dbSNP at a rate of about 90 primarily gene orientated SNPs per month. Based on the observed SNP density in the genome, estimates suggest that the dbSNP dataset may currently represent 20–30% of SNPs in the human genome.

To clarify the scope of dbSNP, the database uses 'SNP' in the looser sense with no requirement or assumption about minimum allele frequency, this presents the intriguing possibility that some as yet undetected disease causing mutations may exist in dbSNP, although the vast majority are likely to be polymorphisms of neutral effect. This is one of the great challenges for genetics, now that we have our snapshot of human genetic variation where lies the disease?

Table 1. SNP and mutation databases and tools on the web

| Tool/Database | URL |
|---|---|
| **Mutation Databases** | |
| OMIM | http://www.ncbi.nlm.nih.gov/Omim/ |
| HGMD | http://www.hgmd.org |
| GDB Mutation Waystation | http://www.centralmutations.org/. |
| HUGO Mutation database initiative | http://www.genomic.unimelb.edu.au/mdi/ |
| **Central SNP databases** | |
| dbSNP | http://www.ncbi.nlm.nih.gov/SNP/ |
| HGBase | http://hgbase.cgr.ki.se/ |
| **Gene Orientated SNP Visualisation** | |
| LocusLink | http://www.ncbi.nlm.nih.gov/LocusLink/ |
| PicSNP | http://picsnp.org |
| CGAP | http://lpgws.nci.nih.gov/ |
| **Tools for SNP visualisation and mapping** | |
| Ensembl | http://www.ensembl.org |
| Golden Path Viewer | http://genome.ucsc.edu/index.html |
| Map Viewer | http://www.ncbi.nlm.nih.gov/cgi-bin/Entrez/hum_srch |
| Genome Database (GDB) | http://www.gdb.org |

Copyright © 2001 John Wiley & Sons, Ltd. 

### The reference SNP dataset (RefSNPs)

The non-redundant reference SNP dataset in dbSNP has been produced by clustering SNPs at identical genomic positions and creating a single representative SNP (designated by an 'rs' ID). This data set considerably streamlines the process of integrating SNPs with other data sources, so only RefSNPs are mapped to external resources or databases. RefSNPs are now closely integrated with other NCBI databases, this has undoubtedly been the key to dbSNP success, variation is now an integral part of the NCBI data infrastructure, so that the biologist can effortlessly browse to dbSNP from diverse NCBI resources, including LocusLink, Mapview and Genbank itself.

Searching dbSNP is possible in a number of ways, including BLAST, text search or via other NCBI tools, eg., LocusLink. Each RefSNP is quite well characterised for the biologist, SNPs mapping to genes are identified and localised to gene regions, eg., introns, exons and promoter regions. Coding SNPs are recorded and amino acid changes are identified. Information derived from all members of the cluster is collated, so for example allele frequencies in more than one population source may be available.

### Candidate SNPs – SNP to assay

The dbSNP data set has one very significant caveat. SNPs generated by both the TSC and human genome sequencing centres were essentially detected by statistical methods to identify 'candidate' SNPs by comparison of DNA sequence traces from overlapping clones [8]. It is important to be aware that these 'candidate' SNPs are mostly of unknown frequency and are unconfirmed in a laboratory assay, this translates to the simple fact that many public SNPs are simply not real or more accurately do not exist at a detectable frequency in a given population. Marth *et al.* (2001) [9] investigated the reliability of these candidate SNPs in some depth, completing two pilot studies to determine how well candidate SNPs would progress to working assays in three common populations. In both studies, they found that between 52–54% of the characterized SNPs turn out to be common SNPs (above >10%) for each population. Significantly, between 30–34% of the characterized SNPs were not detected in each population. These results suggest that if a candidate SNP is selected for study in a common population, there is a 66–70% chance that the SNP will have detectable minor allele frequency (1–5%) and a 50% chance that the SNP is common in that population (>10%). Any genetic study needs to take these levels of attrition between SNP and assay into account (Table 2). There is only one solution to this problem – to determine the frequency of the two million or so public SNPs. This requirement is now widely recognized in the SNP research community and several public groups are seeking to establish large-scale SNP frequency determination projects.

### HGBASE

Although dbSNP is rapidly assuming the position of the primary central SNP database, there is an alternative central SNP database, HGBASE [3]. This database has expanded from its initial remit, as a database of intra-genic sequence polymorphism – to a whole genome polymorphism database. HGBASE encompasses the same classes of variants as dbSNP, indeed HGBASE is a significant contributor to dbSNP and both HGBASE and dbSNP make regular data exchanges to allow data synchronisation, however HGBASE has taken a distinct approach by seeking to summarise all known SNPs as a semi-validated, non-redundant set of records.

HGBASE is seeking to address some of the problems associated with candidate SNPs and so, in contrast to the automated approach of dbSNP, HGBASE is highly curated. The HGBASE curators have carried out the valuable role of identifying SNPs from the literature, particularly older publications before SNP database submission was the norm. The curators are also striving to identify SNP allele frequencies from the literature wherever available and considerable efforts are made to

Table 2. Pitfalls from Candidate SNP to Assay (from Marth *et al.*, 2001)

| SNP to Assay convertion steps | Remaining RefSNPs |
|---|---|
| Reference SNP identified | 2.4M |
| Not Mapped to Human Genome (10%) | 2.16M |
| Assay design not possible or Assay Fails (15%) | 1.84M |
| Not polymorphic in study population (17%) | 1.52M |
| Frequency <20% in chosen population (50%) | 1.26M |
| Common SNPs (>20% freq.) with assay available | 0.63M |

Copyright © 2001 John Wiley & Sons, Ltd. 

relate polymorphisms to human genes, detailing consequences for coding regions, promoters and splice sites. HGBASE currently contains 0.98M human polymorphisms almost all of which are represented in dbSNP (release 12 – Nov 2001). Searching HGBASE is quite simple, tools are available to facilitate BLAST searching and keyword queries. HGBASE is aiming to provide a more-highly validated SNP data set, by filtering out SNPs in repeat and low complexity regions and by identifying SNPs for which a genotyping assay can successfully be designed. This *in silico* quality control approach may be valuable, particularly for the broader community of consumers of SNP data, for the geneticist, HGBASE serves to identify SNPs with a much higher chance of converting from 'candidate SNP' to informative SNP assay. If you take the cost of failed assays into account this is a very valuable objective.

### Tools for SNP visualisation – the genomic context

A powerful alternative interface to public SNP data is offered by the human genome. EnsEMBL and the UCSC Golden Path viewer (Table 1) both maintain current dbSNP and HGBASE annotation on the human genome. User defined queries place SNPs into their full genomic context, giving very detailed information on nearby genes, promoters or regions conserved between species, including mouse and fish. Comparative genome conservation may be particularly useful for analysis of SNP functional impact, as genome conservation is generally thought to be restricted to gene or regulatory regions and so this is one of the most powerful tools for identifying potential regulatory regions or undetected genes [2]. Figure 2 shows Ensembl visualisation of SNPs in the promoter region and first exon of the *PTEN* oncogene, locus visualisation allows immediate assessment of the functional context and conservation of each SNP.

### Tools for SNP visualisation – the gene-orientated context

For the biologist SNP information is generally of most interest when located in genes or gene regions – many tools are now available to visualise such SNPs (Table 1). Almost all NCBI tools integrate

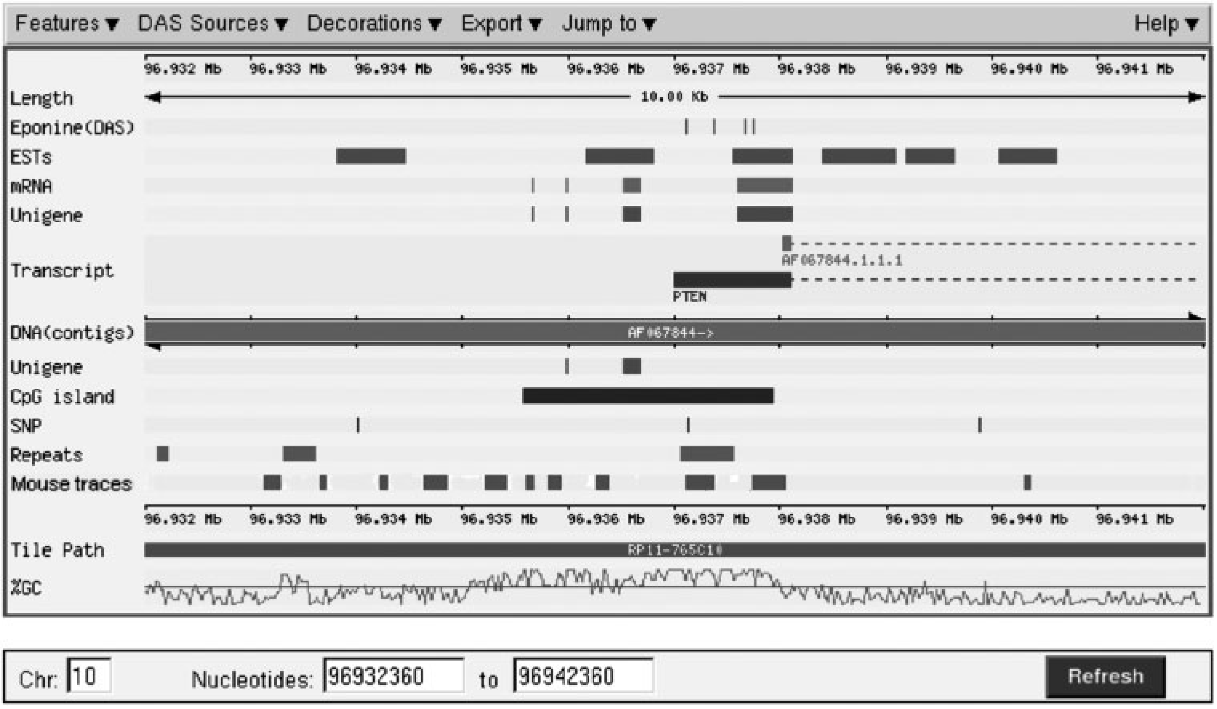

**Figure 2.** Ensembl visualisation of SNPs across the PTEN gene. Reproduced by permission of Ewan Birney

Copyright © 2001 John Wiley & Sons, Ltd. 

directly with dbSNP, for example where SNP data is available, gene entries in LocusLink have a link to a dbSNP RefSNP gene summary (a purple V or VAR link). This summary details all SNPs across the entire gene locus including upstream regions, exons, introns and downstream regions. Non-synonymous SNPs are identified and the amino acid change is recorded, analysis even accommodates splice variants.

Other tools are worth mentioning with different approaches to the presentation of gene orientated SNP data. PicSNP [4] is an interesting tool, which presents a specific catalog of non-synonymous SNPs in human genes. SNPs are further localised to specific gene features, eg., swiss-prot annotated domains, they are also sorted by the functional ontology of genes, so for example it is possible to identify all non-synonymous SNPs in genes with a role in cell–cell signaling.

The CGAP database is also a valuable resource which identifies SNPs by *in silico* prediction from alignments of ESTs, these can be viewed in a JAVA assembly [11]. This information is captured by dbSNP but it is also worthwhile searching CGAP directly, as some potential SNPs which evade detection by the automated SNP detection algorithm can be identified by eye. The JAVA view of trace data makes it possible to confirm the base call of a potential SNP in an EST.

### Mutation databases

The polymorphism data stored in dbSNP is valuable information that helps to define the natural range of variation in genes and the genome but most of the polymorphisms might be assumed to be functionally neutral. By contrast human gene mutation data is functionally defined and has obvious implications for the nature and prevalence of disease and the pathways underlying disease. Many Mendelian disease mutations have been identified since the early 70s and many highly specialised locus specific databases (LSDBs) have been established to collate this data. But in contrast to SNP databases these disparate resources are often unreliably maintained and vary greatly in format and design, effectively making much of this invaluable data unavailable to mainstream biology. Several databases have been established to try to address this situation although mutation data still lacks a comprehensive central database and worse, mutation data is still very poorly integrated with other forms of biological data, particularly the human genome. Hopefully some of the resources below will change this situation.

### The Human Gene Mutation Database (HGMD)

The HGMD was established in April 1996 to collate published germline mutations responsible for human inherited disease. In October 2001, HGMD contained 23345 mutations in 2785 genes. The scope of HGMD is limited to mutations leading to a defined inherited phenotype, including a broad range of mechanisms, such as point mutations, insertion/deletions, duplications and repeat expansions within the coding regions of genes. Recently, HGMD expanded its scope to include disease-associated polymorphisms and so it might be expected to share some overlap with SNP databases. Somatic mutations and mutations in the mitochondrial genome are not included. HGMD invites submissions from researchers but most records are curated directly from mutation reports in more than 250 journals and directly from the LSDBs which are comprehensively linked. To be included, there must be a convincing association of the mutation or polymorphism with the phenotype. All mutations in HGMD are represented in a non-redundant form, unfortunately this does not conserve all mutations constituting a cluster, so it is not possible to determine if mutations are identical by descent, also data is lost on the frequency of mutations. The HGMD search interface is primarily text based, targeted searching tends to rely on knowledge of the correct HUGO nomenclature for a gene.

HGMD contains valuable data, but it is difficult to avoid the feeling that this resource is not fully exploiting its potential, genes are no longer the sole point of reference, instead the whole genomic context of genetic variation is increasingly important. No doubt in an attempt to address this, HGMD has recently entered into a licensing agreement with Celera genomics, providing Celera with a period of exclusive access to new HGMD data. Despite the obvious drawbacks of this agreement for public users, it will undoubtedly enable much needed development of the database. After the period of exclusivity, the new, hopefully much improved, HGMD database will be made publicly available.

Copyright © 2001 John Wiley & Sons, Ltd. 

### Online Mendelian Inheritance in Man (OMIM)

OMIM is not strictly a mutation database, it is more accurately an online catalog of human genes, their associated genetic disorders and Mendelian phenotypes with as yet unidentified genes, based on the long running catalog Mendelian Inheritance in Man (MIM), started in 1967 by Victor McKusick at Johns Hopkins [6]. OMIM is an excellent source of background biology on genes and diseases, it includes information on the most common and clinically significant mutations and polymorphisms in genes. Despite the name, OMIM also covers complex diseases to varying degrees of detail. In October 2001, the database contained over 13090 entries (including entries on 9658 gene loci and 954 phenotypes). OMIM is a manually curated digest of the literature and consequently its entries may not be current and they are not always comprehensive. With this caveat aside it is a very valuable database, with an added bonus of being well integrated with the NCBI database family, this makes movement from a disease to a gene to a locus and *vice versa* fairly effortless. Unfortunately this integration stops short of full sequence integration with human genome viewers such as Ensembl, which is a source of some frustration, but OMIM is nevertheless a highly recommended resource for mutation related data.

### The Genome database (GDB)

GDB is an ambitious genome database, which may soon evolve into the long needed central mutation database – but many difficulties have plagued its development. GDB was established in 1990 as a central repository for mapping information from the human genome project. Throughout the early 90's GDB was the dominant genome database and served as the primary repository for genetic map related information, but in January 98 after several years of uncertain DOE funding, GDB funding was officially terminated. By December 98 funding from another source was found, but at a significantly lower level. By this time other databases had inevitably overtaken GDB as 'central genome databases' [5]. Today GDB is still the most comprehensive source of many forms of genetic data, for example, tandem repeat polymorphisms (it contains over 18000), it also contains extensive information on fragile sites, deletions, disease genes and mutations, collected by a mixture of curation and direct submission. GDB development is ongoing, the database's historical focus on genetic maps is broadening to a more integrated view of the genome ultimately down to the sequence level (which unfortunately is currently lacking). GDB and the human genome organisation (HUGO) are now planning a collaboration to establish federated linkages with 'boutique' LSDBs by setting up a 'Mutation Way Station' to collect and disperse mutations to LSDBs and central databases. This will create a central mutation submission point to provide a consistent interface and a standardised format for all mutation data. After submission to the Way Station an identifier will be assigned and the mutation will be redirected to the appropriate LSDB as well as the central mutation database maintained at GDB. This may be an important strategic move, as the database already contains an unprecedented range of genetic and genomic data, plans to finally integrate a sequence map might well make GDB a prominent resource again. But, this strategy would not be without risks, GDB needs to understand its own strengths and weaknesses, it cannot cover everything. To position GDB as a 'genomic database' would be risky, Ensembl and the UCSC are tough competition as genomic databases, GDB's strengths lie in genetics, so perhaps GDB needs to reposition itself as a 'genetic database' – it's a move that wouldn't even call for a change in initials!

### Conclusions

SNP discovery efforts and genome sequencing data have yielded several million base positions that might be polymorphic in the human genome. The sheer scale of this data offers tremendous opportunities for genetics and biology. But we are now entering a new phase in genetics – the next step is to relate genetic variation to disease. At this point the distinction between polymorphism and mutation data may become less distinct and integration will become a more pressing issue. Human mutation and polymorphism may simply be extremes of a spectrum of disorders. Examples of rare mutations such as homocystinuria mutations with a wider role in complex disease already exist. The human genome presents us with an ideal framework for this data integration, by definition polymorphisms and mutations are essentially sequence-based features, and so the genome is an ideal template for this data. As our understanding of the mechanisms of

Copyright © 2001 John Wiley & Sons, Ltd.

disease increase, the full genomic context of genetic variation may become increasingly significant.

### References

1. Altshuler D, Pollara VJ, Cowles CR, *et al.* 2000. A SNP map of the human genome generated by reduced representation shotgun sequencing. *Nature* **407**: 513–516.
2. Aparicio S, Morrison A, Gould A, *et al.* 1995. Detecting conserved regulatory elements with the model genome of the Japanese puffer fish. *Fugu rubripes*. *Proc Natl Acad Sci U S A* **92**: 1684–1688.
3. Brookes AJ, Lehväslaiho H, Siegfried M, *et al.* 2000. HGBASE: A database of SNPs and other variations in and around human genes. *Nucleic Acids Res* **28**: 356–360.
4. Chang H, Fujita T. 2001. PicSNP: a browsable catalog of non-synonymous single nucleotide polymorphisms in the human genome. *Biochem Biophys Res Commun* **287**: 288–291.
5. Cuticchia AJ. 2000. Future vision of the GDB Human Genome Database. *Human Mutation* **15**: 62–67.
6. Hamosh A, Scott AF, Amberger J, Valle D, McKusick VA. 2000. Online Mendelian Inheritance in Man (OMIM). *Hum Mutat* **15**: 57–61.
7. Kluijtmans LA, van den Heuvel LP, Boers GH, *et al.* 1996. Molecular genetic analysis in mild hyperhomocysteinemia: a common mutation in the methylenetetrahydrofolate reductase gene is a genetic risk factor for cardiovascular disease. *Am J Hum Genet* **58**: 35–41.
8. Marth GT, Korf I, Yandell MD, *et al.* 1999. A general approach to single-nucleotide polymorphism discovery. *Nat Genet* **23**: 452–456.
9. Marth G, Yeh R, Minton M, *et al.* 2001. Single-nucleotide polymorphisms in the public domain: how useful are they? *Nat Genet* **27**: 371–372.
10. Reich D, Cargill M, Bolk S, *et al.* 2001. Linkage disequilibrium in the human genome. *Nature* **411**: 199–204.
11. Riggins GJ, Strausberg RL. 2001. Genome and genetic resources from the Cancer Genome Anatomy Project. *Hum Mol Genet* **10**: 663–667.
12. Risch N. 2000. Searching for genetic determinants in the new millenium. *Nature* **405**: 847–856.
13. Sherry ST, Ward MH, Kholodov M, *et al.* 2001. dbSNP: the NCBI database of genetic variation. *Nucleic Acids Res* **29**: 308–311.

Copyright © 2001 John Wiley & Sons, Ltd. 
